# Mitochondria are specifically vulnerable to 420nm light in drosophila which undermines their function and is associated with reduced fly mobility

**DOI:** 10.1371/journal.pone.0257149

**Published:** 2021-09-03

**Authors:** Jaimie Hoh Kam, Chris Hogg, Robert Fosbury, Harpreet Shinhmar, Glen Jeffery

**Affiliations:** Institute of Ophthalmology, University College London, London, United Kingdom; Biomedical Sciences Research Center Alexander Fleming, GREECE

## Abstract

Increased blue light exposure has become a matter of concern as it has a range of detrimental effects, but the mechanisms remain unclear. Mitochondria absorb short wavelength light but have a specific absorbance at 420nm at the lower end of the human visual range. This 420nm absorption is probably due to the presence of porphyrin. We examine the impact of 420nm exposure on drosophila melanogaster mitochondria and its impact on fly mobility. Daily 15 mins exposures for a week significantly reduced mitochondrial complex activities and increased mitochondrial inner membrane permeability, which is a key metric of mitochondrial health. Adenosine triphosphate (ATP) levels were not significantly reduced and mobility was unchanged. There are multiple options for energy/time exposure combinations, but we then applied single 420nm exposure of 3h to increase the probability of an effect on ATP and mobility, and both were significantly reduced. ATP and mitochondrial membrane permeability recovered and over corrected at 72h post exposure. However, despite this, normal mobility did not return. Hence, the effect of short wavelengths on mitochondrial function is to reduce complex activity and increasing membrane permeability, but light exposure to reduce ATP and to translate into reduced mobility needs to be sustained.

## Introduction

Mitochondria provide much of the energy for cell function in the form of ATP. They have optical characteristics and absorb short wavelength light that is regarded as harmful and is associated with reduced function [1–5]. ATP production requires integrity of the mitochondrial inner membrane, which is where respiratory complexes are located. This membrane has two chromophores, one of which is porphyrin. Porphyrins absorb light at around 420nm and interact with molecular triplet oxygen to form radicals and reactive oxygen species (ROS) that lead to cell death [6–10].There have been numerous studies both in vivo and in vitro examining the impact of short wavelengths [11] but these have never been matched against the light absorbance spectra of mitochondria whose 420nm absorbance dominates the shorter wavelength profiles range. Rather, studies have commonly used a wide range of short wavelengths often from regions where mitochondria are less sensitive. When this is taken in combination with differences in energies and exposure times between separate studies [1,12–14] it is difficult to draw conclusions about how short wavelengths undermine mitochondrial function. However, reduced mitochondrial function impacts negatively on the organism. It not only reduces a key energy source in the form of ATP but also results in an increase in ROS that is a driving factor in ageing and disease.

Here we confirm that mitochondria have a specific absorbance peak at 420nm and that absorbance of this wavelength impacts negatively on their function by assaying for mitochondrial enzyme activities related to complexes I, II, III and IV and the permeability of the inner membrane on which they are located. We also measure changes in ATP and how these translate to changes in fly mobility.

## Materials and methods

### Fly stocks and husbandry

The wild-type, male Drosophila melanogaster Dahomey flies were used throughout. Female files were excluded because they have different metabolism and cycle in their fertility, which may have complicated our results [15–17]. Newly hatched male flies were collected and kept at a density of 30 flies per food vial. They were maintained under standard laboratory conditions and diet with 12/12 hours lighting at 25°C and 70% humidity. Flies were culled by instant freezing with dry ice.

### Light treatment

5 weeks old flies were exposed with 420nm LED light devices (C.H. Electronics, UK) for 15 minutes daily for 7 days at 40W cm^-2^. The half power bandwidth of each was approximately 15nm. The Perspex vials reduced the total energy applied by approximately 3% but had no impact on the wavelength used. In continuous monolayers of freshly killed flies there was a 90% absorption of the 420nm exposure. The experimental group had a matched control (control) whereby they were exposed to the 12/12hr standard laboratory light condition. One more group was added and was exposed to the same light condition as the experimental 420nm LED light but the vials were covered with aluminium foil to black out any light exposure to the flies (Dark control).

### Mitochondrial enzyme activity

Whole body mitochondrial function was measured by enzymatic activities of the complexes (I-IV) in the mitochondrial respiratory chain. 20 flies per group were snap frozen after the 7 days exposure to the lights and homogenised with a pestle in an ice cold homogenising buffer (0.121g of Tris, 0.15g of KCl and 0.038g of EGTA in 50 ml distilled water at pH 7.4. 0.854g of sucrose was freshly added to 10ml of the buffer on the experimental day). The homogenate was then centrifuged at 300 X g for 5 min at 4 ^0^C and the supernatant was collected, aliquoted and stored at -80 ^0^C for the different enzyme activity assays. To standardise the amount of protein in each enzymatic assay, the protein concentration of each sample was measured using the commercial BCA protein assay (ThermoFisher Scientific). For each group, 6 replicates were used in each enzymatic assays, with the activity of each biological replicate estimated from two technical replicate assays.

### Complex I

Complex I (NADH-ubiquinone reductase) was measured using the dichlorophenolindophenol (DCPIP)-couple method. The reaction mix consisted of 25mM potassium phosphate, 3.5g/L bovine serum albumin (BSA), 60 μM DCPIP, 70 μM decylubiquinone, 1 μM antimycine-A, 0.2 mM NADH and 15μg/mL sample protein. Reduction of DCPIP was monitored at 600nm using a spectrophotometer and the reaction was inhibited using 1mM rotenone.

### Complex II

The complex II enzyme activity was measured using the protocol used by Meiklejohn et al. 2013 [18]. Complex II activity was measured by the reduction of DCPIP at 600nm. The reaction mixture contained 30 mM NaH2PO4, 100 μM EDTA, 2 mM KCN, 2 μg/mL antimycin A, 2 μg/mL rotenone, 750 μM BSA, 10 mM succinate, 100 μM DCPIP, 100 μM decylubiquinone and 15 μg/mL sample protein, and the reaction was inhibited with 400 mM malonate.

### Complex III

Cytochrome c reductase (complex III) activity was measured by monitoring the increase in reduced cytochrome c at 550nm. The reaction mix consisted of 25mM phosphate buffer (pH 7.5), 0.5mM KCN, 0.1mM EDTA (pH 7.5), 75 μM cytochrome c, 0.1mM of decylubiquione, 0.025% Tween-20 and 15 μg/mL sample protein, and was inhibited with 5 μg/mL antimycin A. Potassium borohydride was used to reduce decylubiquione. The reaction was stopped using 2 μg/mL rotenone.

### Complex IV

Cytochrome c oxidase (complex IV) was measured using the protocol used by Spinazzi et al. 2012 [19] and the rate of reaction was determined by measuring the rate of oxidation of reduced cytochrome c at 550nm. The reaction mixture contained 50mM phosphate buffer (pH 7.0), 2 μg/ml rotenone, 2 μg/ml antimycin A, 1 mM DDM, 45 μM cytochrome c. Each test contained 15 μg/ml of fly homogenate. The reaction was inhibited with 4 mM KCN. Sodium dithionite was used to reduce cytochrome c.

### ATP

ATP measurements were undertaken using a commercially available ATP determination assay (ThermoFisher Scientific, UK). 5 male flies per group were homogenized in extraction buffer consisting of 6M guanidine-HCL in 100mM Tris and 4mM EDTA, pH 7.8, followed by a heat treatment at 95 ^0^C for 5 min. The homogenates were then centrifuged at 16,000 X g for 15 minutes at 4 ^0^C and the supernatant was collected. The protein concentration of each sample was measured using the BCA assay. For ATP measurement, samples were diluted in 1/10 with the extraction buffer and were added to the reaction mix prepared according to the manufacturer’s instructions.

### Light scattering and passive mitochondrial swelling assay

Mitochondria from 10 male flies per group were isolated and processed for light scattering and swelling according to the procedure used by Navarro et al. (2004) [20]. Flies were homogenised using a pre-chilled mitochondrial storage buffer containing 230mM Mannitol, 70mM sucrose, 1mM EGTA, 10mM HEPES and protease inhibitor cocktail. The homogenate was then centrifuged at 800 x g for 30 min at 4°C and then the supernatant at 11,000 x g for 10 mins to precipitate the mitochondria. Mitochondrial suspensions, containing about 100ug protein/ml was subjected to swelling using a hypotonic solution (90mM KCl, 20mM MOPS-KOH, pH 7.2) and the absorbance changes were measured spectrophotometrically at 540nm for 5 min. The absorption rate (ΔA) and the initial rate (1 min) of mitochondrial swelling (ΔA/min) were recorded. The protein concentration of each sample was measured using the BCA assay (ThermoFisher Scientific).

### Mitochondrial DNA quantification

Mitochondrial suspensions from above were treated with a mitochondria-rupturing hypotonic solution (25mM K2PO4, 5mM MgCl2, pH 7.2) followed by a 3 cycles of freeze-thawing. The freshly obtained fractions were then treated with RNAse A (Thermo Scientific) treatment at 37 ^0^C for 1 hour to remove all RNA. DNA was quantified using a NanoDrop ND-100.

### Negative geotaxis (Locomotor function)

To assess the motor function of the flies, we used the negative geotaxis previously described by Ali et al. 2011 [21]. 100 flies for each group were housed in 10 individual food vial (10 flies per vial) on the day before the assay was performed. The climbing apparatus is made up of 2 empty polystyrene vials vertically assembled with tape. The lower vial was marked with an 8cm mark above the bottom. On the day of the assay, the 10 flies in the vial were transferred to the climbing apparatus and they were allowed to acclimatize to the new environment for 1 min. Then the flies were gently tapped down to the bottom of the climbing apparatus and the number of flies climbing up the 8cm mark in 10 secs were counted. The flies were allowed to rest for 1 min prior to repeating the test. This was done for a total of 10 repeats. The climbing index was determined as a percentage of flies that climbed the 8cm marking relative to the total flies.

### Statistics

Data were analysed with GraphPad Prism v.6.01 (San Diego CA, USA) and statistical analysis was undertaken using one-tailed Mann Whitney U test unless otherwise stated. Data presented are mean ± SEM. The significance was asserted as * p<0.05, ** p<0.01, *** p<0.001.

### Mitochondrial extraction and transmission measurements

A single C57Bl6 mouse was culled by cervical dislocation and the liver was rapidly removed, rinsed and placed into ice-cold mitochondrial storage buffer (230mM Mannitol, 70mM Sucrose, 1mM EGTA, 10mM HEPES). The liver was sliced and homogenised in mitochondrial storage buffer containing protease inhibitor cocktail (Sigma-Aldrich). The homogenate was the centrifuged initially at a low speed of 800 X g for 30 minutes at 4 ^0^C. The supernatant was then removed, and this was centrifuged at 11,000 X g for 10 minutes, the pellet containing the mitochondria was re-suspended in mitochondrial storage buffer.

Transmission spectroscopy was carried out on the extracted mitochondrial sample (dilution 1:1) in a 1cm plastic cuvette using a Maya2000Pro spectrometer (200–1100nm, OceanOptics) using two lamps to cover the entire wavelength range. The UV range was observed with a deuterium lamp (DH-Mini, OceanOptics) and the longer wavelengths with a quartz-halogen source (HL-2000, OceanOptics). An identical cuvette filled with buffer solution was used as the reference source. Repeated measurements with both lamps were made over a period of two hours following sample preparation. During this period, the particulate component of the sample was settling within the cuvette, allowing increased transmission, especially at the shorter wavelengths.

## Results

**Fig 1:**
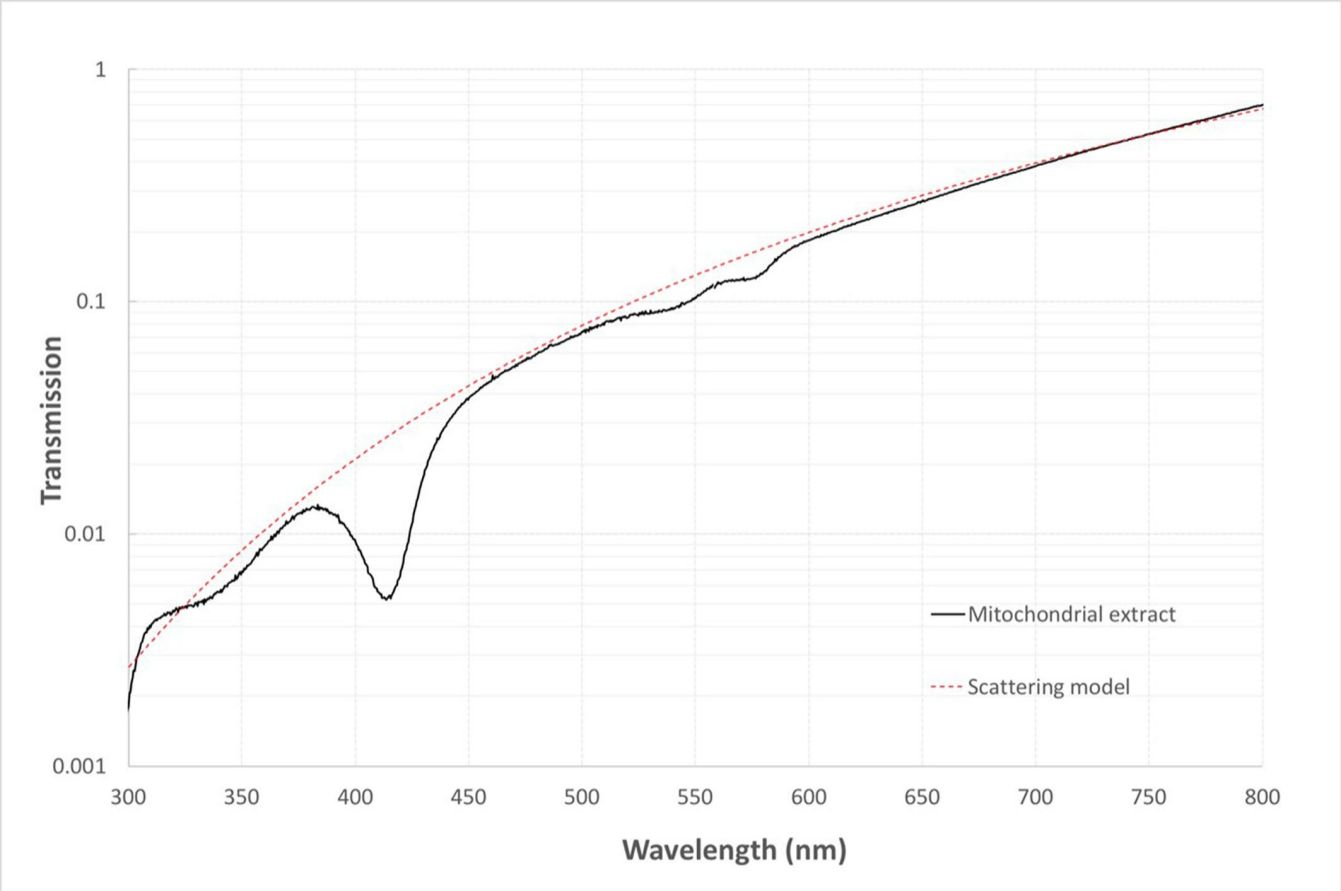
Shows the 1cm cuvette sample transmission spectrum (black line) from two separate measurements of mitochondrial transmission taken from extract of mouse liver made using a deuterium and quartz-halogen lamp. The longer wavelength spectrum has been aligned in transmission with the UV measurement to account for the particulate settling in the sample. The dashed red line shows a simple wavelength power-law scattering model that can be used to correct the spectrum for this absorption-band free spectral component.

The spectral transmission of mitochondria has been published previously, but this study is not accessible to many [22]. Fig 1 shows our transmission measurements, which are in very close agreement with those of Nakajima et al. [22]. Mitochondria have reduced transmission at shorted wavelengths particularly in the ultra-violet and specifically at 420nm. Having confirmed a key absorbance at 420nm we use this as our selected wavelength for exposures to examine its impact on mitochondrial function. We initially expose for 15 mins at 40mW/cm^2^ to be consistent with other studies we have undertaken in light exposures to modify mitochondrial function in drosophila [23,24].

**Fig 2:**
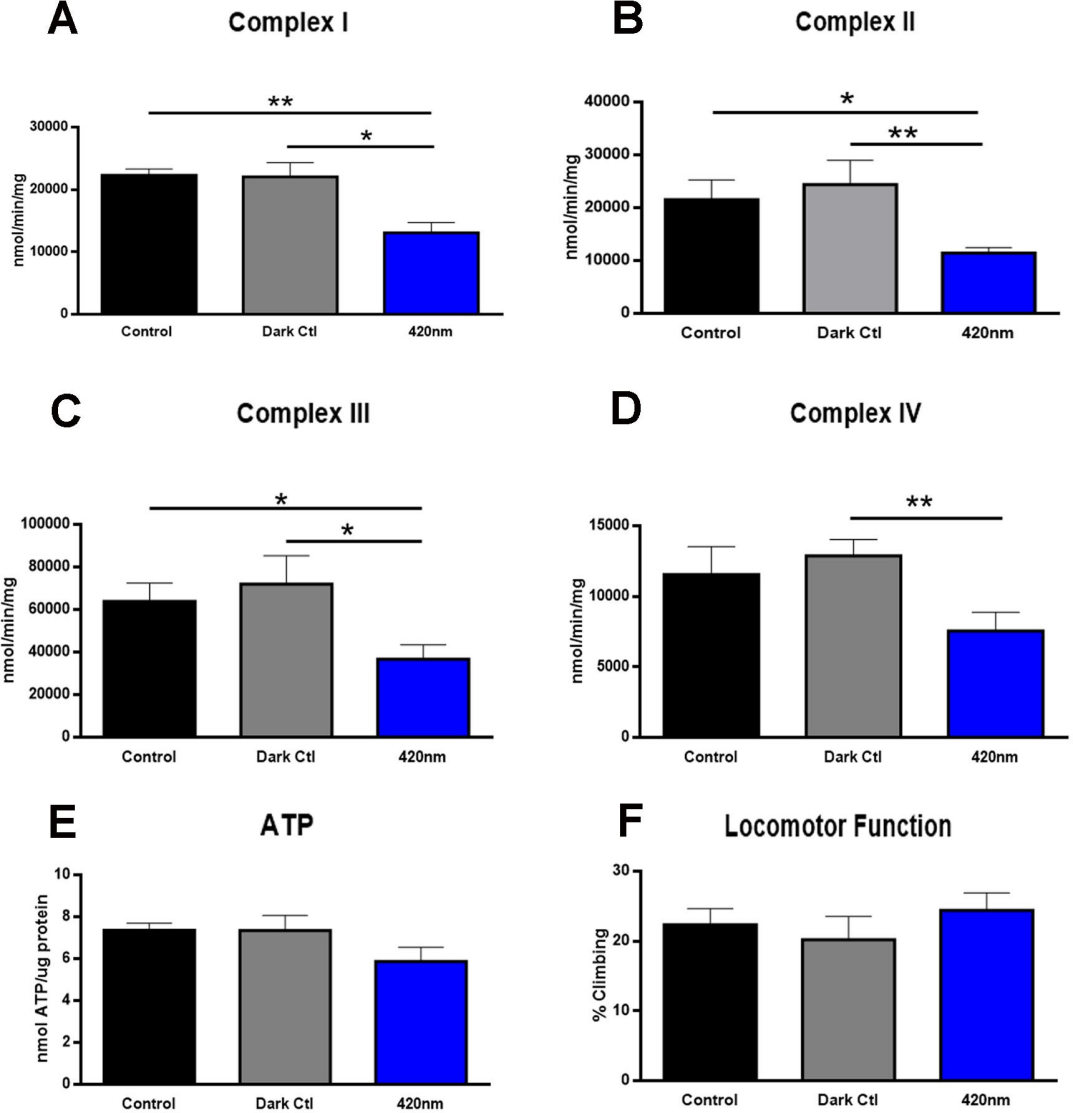
Mitochondria complex activity (A-D) for I, II, III and IV was measured after 420nm (Blue) light exposure for 15 mins daily over 1 week. Blue histograms are vials exposed to 420nm LED light for 15 mins daily for 7 days. Black histograms represent controls where vials were exposed to 12/12h standard light condition and grey histograms are vials were shielded from light by aluminium foil. 420nm significantly reduce the activity of each complex when compared to both controls (A-D). Level of ATP (E) was reduced with 420nm, but this was not significant. Locomotor function (F) did not change for 420nm treated flies. Hence 420nm significantly impacted on mitochondrial complex activities, but this did not translate to reduced function of the organism. Abbreviations: * p<0.05, ** p<0.01.

Daily 420nm exposures significantly reduced activity of each of the four mitochondrial complexes examined. Reductions were of the order of 50% in each. However, although associated ATP levels were reduced by approximately 20%, this did not reach statistical significance. There were no changes in mobility (Fig 2).

**Fig 3:**
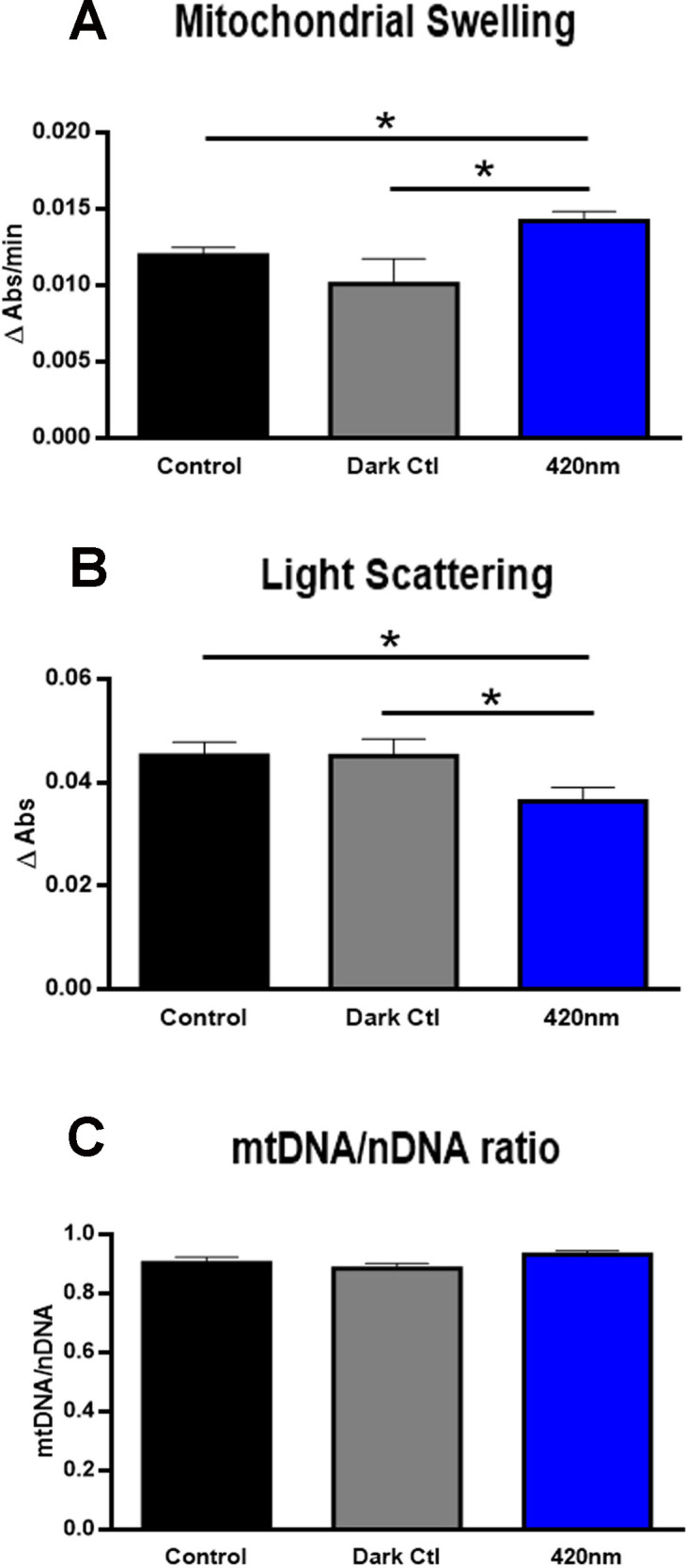
Mitochondrial membrane permeability and DNA content. Mitochondrial membrane permeability was determined by hypotonically induced mitochondrial swelling and the absorption changes were followed spectrophotometrically at 540nm [20,31]. Swelling was measured in each of the light exposed groups (A). Mitochondrial swellings significantly increase in response to 420nm. A direct estimation of mitochondrial size is obtained by light scattering. Mitochondria scattering light (B) was significantly reduced following exposure to 420nm. Damaged mitochondria in 420nm swell more than mitochondria in control group. The bigger the mitochondria, the less likely the light is scattered as it is being absorbed. Exposure to 420nm light did not impact on mitochondrial DNA content (C). Controls in black are exposed to 12/12h standard light condition and grey are vials shielded from light with aluminium foil. Abbreviation: * p<0.05. nDNA stands for nuclear DNA.

Mitochondria lose their ability to regulate their inner membrane permeability with age or in some diseases [25–30]. Here we show that mitochondria exposed to 420nm swell when hypotonically induced (Fig 3A). This dynamic increase of mitochondrial inner membrane permeability induces collapses of transmembrane ion gradients, resulting in membrane depolarisation and uncoupling of oxidative phosphorylation [29]. This is likely associated with the decrease in the activities of the mitochondrial enzyme complexes that are located on this membrane and may contribute to a potential reduction of ATP synthesis.

A measure of mitochondrial size related to the swelling in flies exposed to 420nm light is by changes in light scatter at an absorbance of 540nm measured spectrophotometrically [20,32]. The results show that mitochondria from 420nm treated flies had significantly reduced scatter consistent with their increased size compared to controls (Fig 3B). Furthermore, the results also showed that the passive rate of mitochondrial swelling in a hypotonic medium was significantly faster in the 420nm treated flies (Fig 3A)

Short wavelength light exposure is associated with DNA damage, although this is mainly confined to ultra violet (UV) absorption [33]. However, reduced mtDNA content following 420nm exposure would indicate an additional mechanism of damage rather than one associated with restrictions in complex activity [34,35], but 420nm exposure did not change mtDNA content (Fig 3C).

There are an infinite series of energy/time combinations that could be used to further investigate the impact of 420nm on mitochondrial performance. However, there is evidence that mitochondrial complex activity has the ability to rebound rapidly following restricted insults [12]. This may result in limited restriction to ATP synthesis and fly mobility following 15 min exposures [36]. Hence, we progressed by giving long single exposures of 3h and monitored how long the effect of 420nm on the mitochondrial integrity and function last, when sampled for 72h for key metrics.

**Fig 4:**
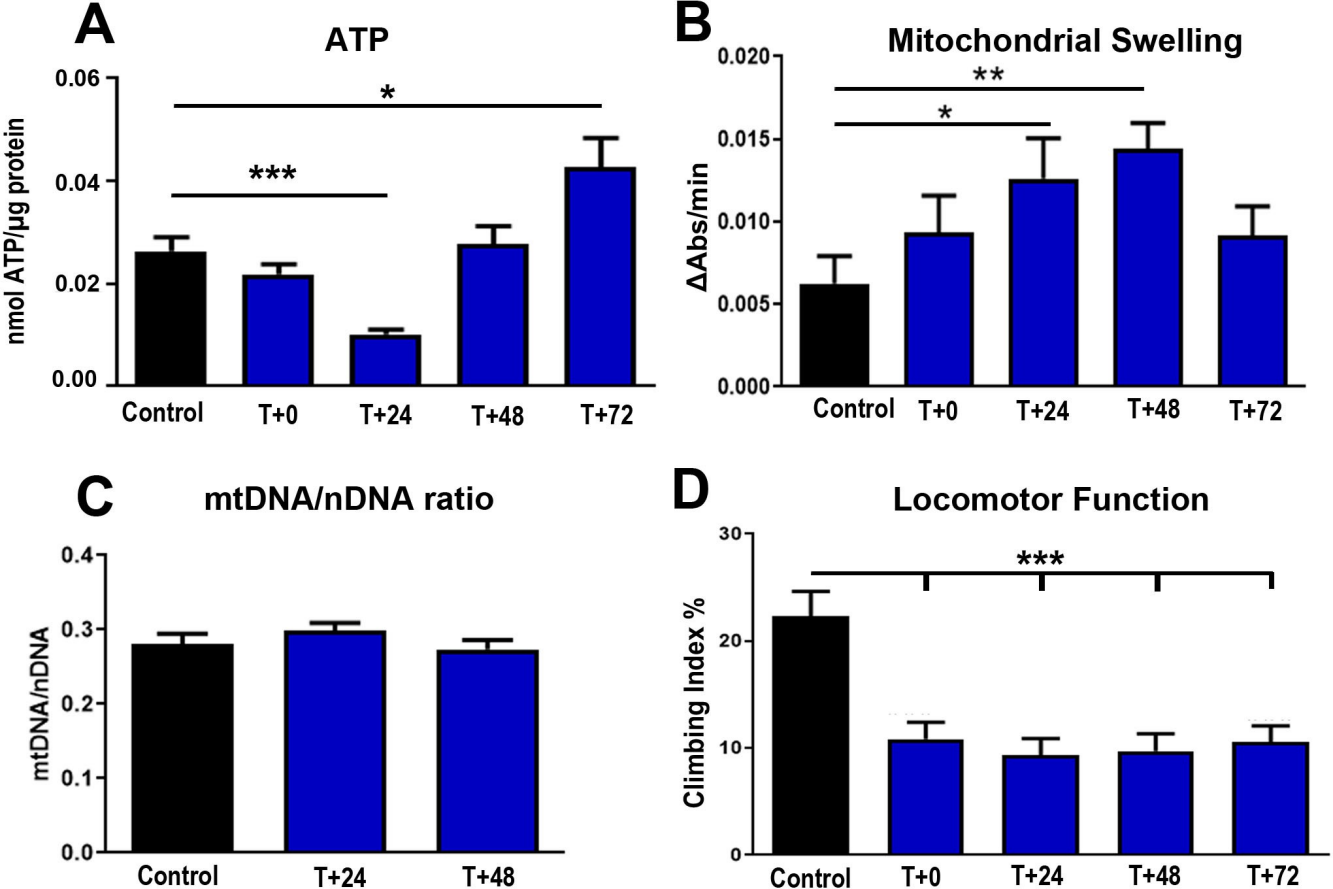
Three hours 420nm light reduced ATP significantly at 24h but there was a strong bounce back from this, significantly increasing levels found at 72h over controls that were not exposed to 420nm light (A). Similar patterns are found with mitochondrial hypotonic swelling, which increased progressively over 48h, but then declined to within the normal range (B). The longer exposure did not change mitochondrial DNA content (C). There is a significant reduction in climbing ability of >50%, which did not recover when animals were re-tested at 24, 48 and 72h post exposure (D). Abbreviations: * p<0.05, ** p<0.01, *** p<0.001.

These extended exposures did not have an immediate impact on ATP, but had a significant impact reducing ATP levels by approximately 60% at 24h post exposure. However, there was a subsequent rebound bringing it back to its pre-exposure level by 48h (Fig 4A). The rebound continued at 72h with a significant elevation in ATP over controls. Mitochondrial hypotonic swelling significantly increased by approximately 100% at 24h post exposure which matches the time point when ATP is significantly reduced, and remained elevated at 48h, returning towards normality at 72h post exposure (Fig 4B). These mitochondrial changes appear to be independent of any change in mtDNA, which remained unaltered (Fig 4C).

Three hour exposures to 420nm were associated with a significant reduction in fly mobility that showed no recovery over 72h. At each of the 4 time periods examined, mobility was significantly reduced by >50% with no indication of improvement. Hence, while ATP and mitochondrial membrane permeability recovered, the absence of appropriate mitochondrial function over this period resulted in sustained damage to mobility.

## Discussion

Understanding the effects of blue light has become an increasingly important health issue as we are exposed to increasing levels of short wavelengths over the day via computer screens and artificial lighting that is LED based. Here, we confirm a key reduction in mitochondrial light transmission at 420nm and use this wavelength to determine its impact on mitochondrial integrity and function through to fly mobility. Short 420nm exposures daily over a week significantly reduced mitochondrial enzymes complex activities and increased mitochondrial membrane permeability but did not translate to significantly reduced ATP or mobility. This may be because the periods between exposures allowed mitochondrial recovery [12,13]. When a longer single exposure of 3h was used, ATP, mitochondrial membrane permeability and fly mobility were significantly impaired. But over 72h, ATP and mitochondrial swelling showed extended recovery, but fly mobility did not.

Mitochondrial absorbance at 420nm is likely due to porphyrin, which absorbs strongly in the Soret band that peaks around 420nm. It is possible that the effects of 420nm are mediated by protoporphyrin IX, the immediate precursor in haem synthesis, which is known to be photo-active in the production of singlet oxygen and related ROS [37–41]. Porphyrin excitation via 420nm may limit oxygen transport undermining oxidative phosphorylation and hence mitochondrial function. Porphyrin is also a key element in haemoglobin. In this context it may be relevant that blue light exposure to the human body results in decreasing systolic blood pressure and increasing heart rate. It also increases blood flow [42].

While many studies have examined the impact of short wavelengths, few have specifically used 420nm, a wavelength that mitochondria specifically absorb. Without wavelength standardisation it is difficult to evaluate the impact of short wavelength light in different studies. Further, many have use different exposure times. But the significance of 420nm in the blue light spectrum is highlighted by Marie et al. [43]. Using an *in vitro* cell preparation, they systematically shifted wavelength exposures from 390nm to 520nm in 10nm steps measuring changes in mitochondrial membrane potential, oxidative stress, and mitochondrial damage. When tissues were stressed, they found that for each metric 420nm had the greatest impact.

Daily 15 min exposures, did not translate to a significant reduction in ATP or a change in fly mobility. This is likely due to recovery between exposures. But this changed when exposures were lengthened to 3h. Here ATP was reduced for 24h before recovering and subsequently becoming elevated. A similar pattern is seen with cytochrome oxidase activity following short wavelength exposure. Using the same exposure time as us, Chen et al. [12] exposed rats to 404nm and subsequently charted the activity of complex IV in the retina. Their data are like ours. However, they then measured activity on progressive days revealing a 250–300% increase in Cytochrome C oxidase over controls 1–6 days later. This is similar to our increase in ATP at 72h. Cytochrome oxidase activity in their study only returned to control levels 14 days after exposure. Hence, there is a marked compensatory period of complex activity and ATP production following short wavelength exposure.

There are likely to be many changes that result from long or short wavelength exposure to mitochondria and an obvious metric is a change in their morphology. This has not been extensively examined over longer time periods. However, in *in vivo* real time imaging of labelled mitochondria we found no evidence of changes in morphology or mitochondrial dynamics in a 3h period. Whether this remains true over the longer period remains to be resolved.

The 3h exposures resulted in reduced fly mobility that did not recover even though ATP increased above controls. The apparent elevation may have resulted from normal production but with reduced consumption due to impaired physiology. In support of this, it has been established that impaired mitochondrial energy production alone can directly lead to dysfunction and cell death [44]. However, a key critical comment in relation to our study that we did not control for was the possibility that mobility may return if examined over a longer time period. Because we had no idea when this may happen, the potential experiments to resolve this could be open ended and were not explored further.

We have exposed whole flies to 420nm, but mitochondrial distribution and density varies across the body. Hence, we do not know how mitochondria respond to light in different tissues. This remains a significant absence. Perhaps in tissues with low mitochondrial density, the impact of light many be relatively reduced. However, because of its unique exposure to light and its very high mitochondrial concentration, the retina is a key target for needing to understand the impact of short wavelengths. Here blocking blue light following bright white light exposure in rodents proved protective [45]. Further, in human psychophysical experiments on colour matching and adaptation, very long recovery times are found when short wavelength light is used, but not with longer wavelengths [46]. It is possible that such effects result from the impact of short wavelengths directly upon retinal mitochondria that in turn modulate psychophysical adaptation.

An important facet of blue light exposure is the implications it has for human health in modern artificial environmental lighting. There have been major shifts in environmental lighting away from incandescent bulbs that generate a continuous spectrum towards LED units that generate a limited pattern of wavelengths that appear white to the human eye. Many of these contain significant peaks in the short wavelength range including 420nm [47]. Given the amount of time spent under such lighting, it may be important to ask what the consequences could be for human health based on mitochondrial modulation.
